# Let’s Stop Spinning Our Wheels: Strengthening the Case for Community-Engaged Transportation Solutions

**DOI:** 10.1093/geroni/igaa057.2461

**Published:** 2020-12-16

**Authors:** Holly Dabelko-Schoeny, Noelle Fields, Nina Silverstein

## Abstract

On average in the United States, older adults outlive their ability to drive by seven years. Having safe, affordable and accessible alternative transportation options is critical to supporting the well-being of older adults and their ability to age in community. This symposium will provide evidence for utilizing community-engaged research strategies with diverse populations to identify the opportunities and barriers for the development and utilization of alternative transportation. The presentations will include up-to-date examples of innovative alternative transportation solutions and evaluation data. The first presentation will illustrate how community-based participatory research (CBPR) strategies were used to develop, use and evaluate alternative transportation options including walking, biking, fixed route busing, senior circulator, ride sharing and transit training in an age-friendly community. Data were collected through mapping the built environment, an electronic daily transportation diary app called “MyAmble” on tablets, walk audits and focus groups. The second presentation uses an environmental justice (EJ) framework to present the findings of a qualitative interpretive meta-synthesis (QIMS) about older adults’ experiences with outdoor spaces and buildings and transportation as part of the World Health Organization’s age-friendly network assessment. The third paper explores the barriers and facilitators of transportation among diverse older adults (English, Nepali, Khmer, Somali, Russian and Mandarin Chinese) using Rapid and Rigorous Qualitative Data Analysis (RADaR) technique. The final paper examines the challenges of rural transportation services supported by senior centers. The symposium will conclude with a critical reflection on the empirical contributions needed to advance scholarship on alternative transportation for older adults.

